# A comparative analysis of low intensity ultrasound effects on living cells: from simulation to experiments

**DOI:** 10.1007/s10544-022-00635-x

**Published:** 2022-10-24

**Authors:** Giulia Tamboia, Michele Campanini, Veronica Vighetto, Luisa Racca, Luca Spigarelli, Giancarlo Canavese, Valentina Cauda

**Affiliations:** Department of Applied Science and Technology, Politecnico di Torino, Corso Duca degli Abruzzi 24, 10129 Turin, Italy

**Keywords:** Acoustic cavitation, Nanoparticles, Reactive oxygen species, Acoustic field simulation, Sonochemiluminescence, Electron paramagnetic spectroscopy

## Abstract

Ultrasounds are already broadly exploited in clinical diagnostics and are now becoming a powerful and not harmful tool in antitumoral therapies, as they are able to produce damages towards cancer cells, thank to inertial cavitation and temperature increase. The use of US alone or combined to molecular compounds, microbubbles or solid-state nanoparticles is the focus of current research and clinical trials, like thermoablation, drug sonoporation or sonodynamic therapies. In the present work, we discuss on the non-thermal effects of ultrasound and the conditions which enable oxygen radical production and which role they can have in provoking the death of different cancer cell lines. In this perspective, we set a mathematical model to predict the pressure spatial distribution in a defined water sample volume and thus obtain a map of acoustic pressures and acoustic intensities of the applied ultrasound at different input powers. We then validate and verify these numerical results with direct acoustic measurements and by detecting the production of reactive oxygen species (ROS) by means of sonochemiluminescence (SCL) and electron paramagnetic resonance (EPR) spectroscopy, applied to the same water sample volume and using the same US input parameters adopted in the simulation. Finally, the various US conditions are applied to two different set of cancer cell lines, a cervical adenocarcinoma and a hematological cancer, Burkitt’s lymphoma. We hypothesize how the ROS generation can influence the recorded cell death. In a second set of experiments, the role of semiconductor metal oxide nanocrystals, i.e. zinc oxide, is also evaluated by adding them to the water and biological systems. In particular, the role of ZnO in enhancing the ROS production is verified. Furthermore, the interplay among US and ZnO nanocrystals is evaluated in provoking cancer cell death at specific conditions. This study demonstrates a useful correlation between numerical simulation and experimental acoustic validation as well as with ROS measurement at both qualitative and quantitative levels during US irradiation of simple water solution. It further tries to translate the obtained results to justify one of the possible mechanisms responsible of cancer cell death. It thus aims to pave the way for the use of US in cancer therapy and a better understanding on the non-thermal effect that a specific set of US parameters can have on cancer cells cultured *in vitro*.

**Graphical abstract:**

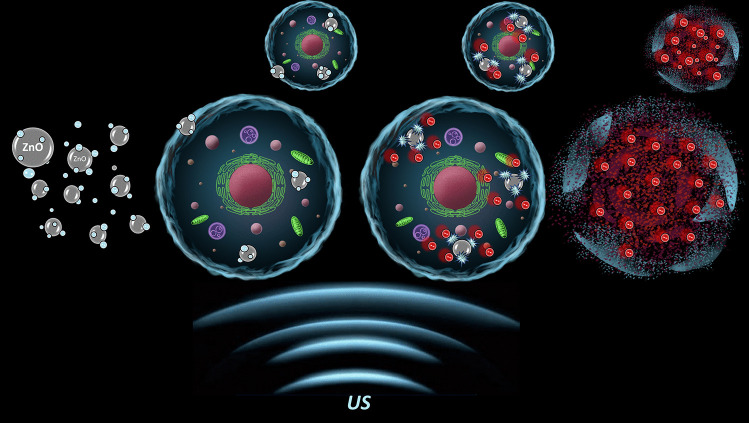

**Supplementary Information:**

The online version contains supplementary material available at 10.1007/s10544-022-00635-x.

## Introduction

Ultrasound (US) is a mechanical pressure wave propagating longitudinally in a continuous medium and characterized by frequencies higher than the upper audible limit (approximately 20 kHz) (Shibagichi et al. 2011). It is usually generated by an ultrasonic transducer, typically made of piezoelectric material, which is excited at the proper frequency by a generator and it is able to transform electric signal into mechanical displacement (Canavese et al. 2018). Transmission of ultrasound wave in fluids can lead to thermal effects, mainly an increase in temperature, but also to non-thermal ones, which can be considered a complex set of processes, including microstreaming, radiation forces and acoustic cavitation (Canavese et al. 2018). Acoustic cavitation is a phenomenon that involves formation and growth of gas bubbles (Shibagichi et al. 2011), caused by the time-varying sinusoidal pressure cycles applied to the liquid, corresponding to expansion and contraction phases. Two possible conditions can be distinguished: stable cavitation, which is characterized by permanent bubbles that oscillate for many cycles of pressure variation, and transient or inertial cavitation. In this last condition, the gas bubbles expand enormously with respect to their original size and then violently collapse releasing a large amount of energy (Neppiras 1980). During such collapse, very high temperatures (above 5000 K) and pressures (above 800 atm) are generated locally causing the ionization of gas, microjet streaming, and thermal dissociation of water molecules into hydroxyl radicals (HO·) and hydrogen atoms (H·), the cleavage of dissolved oxygen, O_2_, leading to the formation of several reactive oxygen species (ROS) (McMurray and Wilson 1999). If inertial cavitation happens in a biological environment, the result, depending on the amount of generated ROS, can lead to a highly oxidative stress environment, that is fatal for cells, including cancer ones (Shibagichi et al. 2011). Within the different radicals, the hydroxyl one is considered one of the strongest, due to its highest reduction potential and its capability to react with several biomolecules (Vighetto et al. 2019). It is therefore highly desired to effectively apply US inducing the ROS generation, specifically towards cancer cells, with the final aim to maximize their death and achieve an efficient cancer therapy. The sonodynamic therapy has indeed the above-mentioned aim and is typically assisted by organic molecules, called sonosensitizers, allowing to maximize the effect of US (Shibagichi et al. 2011; Canavese et al. 2018). Although already used in some clinical asset to treat cancer, molecular sonosensitizers are typically hydrophobic molecules and non-specific to cancer tissues.

The recent advancement in research has proposed the use of various types of nanoparticles, which can be better dispersed in water-based media and can be efficiently targeted to cancer cells (Racca and Cauda 2020). The mechanism of interaction among ultrasound and organic or inorganic nanoparticles (NPs) in water solution exposed to such acoustic irradiation can lead to different effects. Depending on their nature, NPs of organic polydopamine or inorganic one like cerium oxide can scavenge the produced ROS from water, reducing the oxidative stress (Carmignani et al. 2022; Battaglini et al. 2020). On the other hand, semiconductor nanoparticles like zinc oxide or titania (Matos et al. 2020) can support the ROS generation, typically lowering the inertial cavitation threshold. In particular, it is supposed that their surface roughness and porosity, as well as the presence of organic functional groups imparting a hydrophobic milieu to their surface, can immobilize nanosized gas bubbles and provide nucleation sites for their cavitation (Ancona et al. 2020; Lops et al. 2019). Furthermore, the emission of photon in the ultraviolet–visible region during inertial cavitation is supposed to photoexcite large band gap semiconductor NPs, such as ZnO and TiO_2_ (Vighetto et al. 2022). This light excitation produces an electron–hole separation in their band structure and both electrons and holes can react with oxygen and water molecules adsorbed at the semiconductor surface, transforming them in free radicals.

Several groups have applied different types of NPs as sonosensitizer or, more in general, as acoustically-activated nanomaterials under ultrasound stimulation at low intensity, towards a therapy called sonodynamic (SDT) (Racca and Cauda 2020; Canaparo et al. 2020). The use of SDT with solid-state NPs is still under study, due to the poor comprehension of the mechanisms under which NPs and ultrasound synergistically cooperate to induce cell death or spare healthy tissues and to possible side effect on the NPs use. The possible mechanisms of cell death induced by ultrasound and in particular by NP-assisted ultrasound are manifold and depends on the US figures of merit and on the type and nature of the nanomaterial used (Racca and Cauda 2020). Very generally, both thermal and non-thermal effects of US can induce toxicity and even death on the cancer cell. The presence of NPs can then increase temperature and ROS production, while the sonoluminescence can activate NPs to produce radicals and other toxic species. Furthermore, NPs motion in the US field can create mechanical injury to cells, and also ion release should be considered, as well as electric or even piezoelectric stimulations with consequent nanomaterial polarization and cell charge imbalance (Racca and Cauda 2020).

In the present study, we aim to advance the knowledge about low-intensity ultrasound triggering ROS production and non-thermal effects, simplifying the problem to a cylindrical water volume contained in a well. In particular, we hypothesize that it is possible to simulate the acoustic field in a well-defined and controlled set-up and validate such model with direct acoustic measurements and with the detection of indirect effects, like the production of ROS induced by inertial cavitation.

We first simulated the acoustic field of the water to properly estimate the US field intensity and pressure across the water solution, with spatial resolution throughout the sample volume. For this purpose, simulations were implemented in COMSOL^®^ Multiphysics, as previously reported in the literature (Hasnul Hadi et al. 2019) and by some of us (Spigarelli et al. 2020).

Secondly, we carried out experimental studies to validate the numerical simulations, directly measuring the acoustic pressure inside the sample well, and we detected through sonochemiluminescence (SCL) (Tiong et al. 2017) and Electron Paramagnetic Resonance (EPR) spectroscopy the production of ROS in water solution at the previously simulated conditions. Clearly, the simulation set-up was maintained as much close as possible to the experimental one, so that a clear comparison can be made. Previous studies were reported in the literature trying to compare the simulated pressure field with experimental data obtained from US transducers in a low frequency range. More in details, these works proposed to compare either the simulated pressure field with acoustic pressure measurements (Wei and Weavers 2016; Fontana et al. 2021) or with indirect effect caused by US, like the SCL emission (Hasnul Hadi et al. 2019).

In the present paper, the obtained simulation and experimental measurements of acoustic pressure and ROS were then combined with biological data, i.e. the measurement of cell viability of two different cancer cell lines, treated with the same ultrasound irradiation parameters. In particular, a cervical adenocarcinoma (KB cell line) and a Burkitt’s lymphoma (Daudi cell line) were used to investigate the effect of US on two different biological cell systems and more in details to verify the cytotoxic role of the generated ROS under the application of specific US parameters.

As previously reported (Alassaf et al. 2013; Leskinen and Hynynen 2012; Snehota et al. 2020), the US exposure of an *in vitro* cell culture system has many parameters which have to be controlled in order to render the experiments reproducible and also comparable to other literature results. For example, US can be easily altered by varying the coupling medium, the cell culture vessel and medium, as well as by sample rotation, movement, and reflection of US from the vessel wall. In our study, we have maintained the same experimental conditions applied in the above-mentioned acoustic measurement and ROS generation experiments, as well as used in the simulation set-up. In this way, we assume it should be possible to compare the data obtained from these different experiments and drive uniform conclusions.

To further advance the knowledge in the field of nanomedicine, semiconductor zinc oxide nanocrystals (ZnO NCs) stabilized with amino-propyl functional groups were considered in the presented system, in view also of the previous literature results (Vighetto et al. 2019, 2022; Ancona et al. 2020; Racca et al. 2020; Garino et al. 2019). A possible enhancement in ROS production was evaluated from these NCs in combination with US at various power densities and treatment times. Furthermore, ZnO-NH_2_ NCs were also administered to cell in a sub-toxic concentration, in order to investigate their possible synergistic effects related cell death.

The proposed evaluations aim thus to shed light on the effect of ultrasound in a living system *in vitro* and increase the level of knowledge on the different key parameters to help the advancement in the field of physical therapies and nanotherapeutics against cancer.

## Materials and methods

### Acoustic field simulations

The acoustic field was induced in 2 mL of a water solution contained in a well and simulated with COMSOL^®^ Multiphysics (version 5.5, COMSOL Inc.), using the *Pressure Acoustic Frequency domain* interface. By means of this interface, the linearly approximated form of the acoustic wave equation for a time-harmonic pressure wave was solved for the specific frequency (*f*) of 1 MHz, the same applied with the transducer in the experiments.

As done in previous works (Spigarelli et al. 2020; Tiong et al. 2017; Lei et al. 2013), some assumptions were implemented: (1) all the thermoviscous effects were neglected: the simulated system was adiabatic, with an equilibrium temperature equal to the standard ambient one (298.15 °K); (2) the fluid (water) was homogeneous, isotropic, in quiescence and unperturbed from any background pressure field; (3) the US wave propagated linearly through the fluid; (4) the presence and the generation of cavitation bubbles in the fluid was not simulated; (5) the system was simulated by using the COMSOL^®^ *Viscous Fluid* model.

Therefore, the acoustic wave equation solved by the software for the acoustic pressure field *p* had the following form (Eq. 1) (Lei et al. 2013):

1 $$\nabla \cdot \left(-\frac{1}{{\rho }_{c}}(\nabla p-{{\varvec{q}}}_{d})\right)-\frac{{k}_{eq}^{2}{p}}{{\rho }_{c}}={Q}_{m}$$

where ${k}_{eq}= \frac{\omega }{{c}_{c}}$ is the wave number, equal to the ratio between the wave angular frequency *⍵* = 2*πf* and the complex speed of sound in water ${{c}_{c}=c(1+i\omega \frac{\delta }{{c}^{2}})}^{0.5}$, whose real part is represented by *c* = 1497 m/s (Snehota et al. 2020), while the imaginary part, which takes into account the damping of the US wave due to viscous losses, contains the sound diffusivity $\delta =\frac{1}{\rho }(\frac{4}{3}\mu +{\mu }_{b})$, where $\rho$ is the water density, *μ* is the water dynamic viscosity and *μ*_*b*_ is the water bulk viscosity. Then, ${\rho }_{c}=\frac{\rho {c}^{2}}{{c}_{c}^{2}}$ is the complex water density, ${q}_{d}$ and ${Q}_{m}$ are the dipole and the monopole domain source respectively, both of them set to 0 since no kind of source was assumed to be present inside the system, and *p* is the acoustic pressure, computed by the software at each point of the domain.

Since only the fluidic domain was included in the simulations, the polystyrene walls of the well were modeled by implementing at the lateral surface of the domain the *Impedance* boundary condition (Comsol 2020) (Eq. 2), where the value of the polystyrene acoustic impedance (${Z}_{ps}={\rho }_{ps}\cdot {c}_{ps})$ was set in the right-hand side of the equation; the same kind of boundary condition, this time using the air acoustic impedance (${Z}_{air}={\rho }_{air}\cdot {c}_{air})$, was imposed at the domain top surface (Eq. 3), in order to model the contact of the upper surface of the solution with the air. The sinusoidal displacement generated by the oscillating US transducer at the bottom basis of the fluid was modeled by adding the *Normal Displacement* boundary condition (Comsol 2020) (Eq. 4) at the domain bottom surface: the displacement amplitude (L_D_), inserted on the right-hand side of the equation, was determined from the intensity value of the incident wave to the system (${I}_{0}$), defined according to Eq. 5 (The Acoustic Bubble n.d.). This value was calculated by considering the exponential reduction of the US intensity caused by the wave propagation from the transducer (generating its acoustic intensity I_input_) through the 1.3 mm thick polystyrene basis (t) of the well, characterized by its attenuation coefficient α (Eq. 6) (The Acoustic Bubble n.d.), considered at 298.15 K and US frequency of 1 MHz.

2 $$\begin{array}{cc}-{\bf{n}}\cdot \left(-\frac{1}{{\rho }_{c}}\left(\nabla p-{{\varvec{q}}}_{d}\right)\right)=-\frac{pi\omega }{{Z}_{ps}}& \mathrm{on\:the\:lateral\:surface},\end{array}$$

3 $$\begin{array}{cc}-{\bf{n}}\cdot \left(-\frac{1}{{\rho }_{c}}\left(\nabla p-{{\varvec{q}}}_{d}\right)\right)=-\frac{pi\omega }{{Z}_{air}}& \mathrm{on\:the\:top\:surface},\end{array}$$

4 $$\begin{array}{cc}-{\bf{n}}\cdot \left(-\frac{1}{{\rho }_{c}}\left(\nabla p-{{\varvec{q}}}_{d}\right)\right)={(i\omega )}^{2}{L}_{D}& \mathrm{on\:the\:bottom\:surface},\end{array}$$

5 $${I}_{0}=1/2\rho c{\omega }^{2}{{L}_{D}}^{2}\to {L}_{D}= \sqrt{\frac{2{I}_{0}}{\rho c{\omega }^{2}}}$$

6 $${I}_{0}= {I}_{input}{e}^{-2\alpha t}$$

where **n** is the outward normal vector to the domain surface.

The domain was discretized with the COMSOL model of *Mapped* quadrilateral mesh in the boundaries, which was reproduced even at each point of the bulk by applying the *Swept* discretization method. The maximum mesh element size was imposed equal to 1/8 of the wavelength of the applied US, and the minimum one to 1/10, since 8–10 mesh elements within the wavelength are enough to simulate acoustic fields (Lei et al. 2013), providing a sufficiently accurate solution of the acoustic wave equation.

**Fig. 1:**
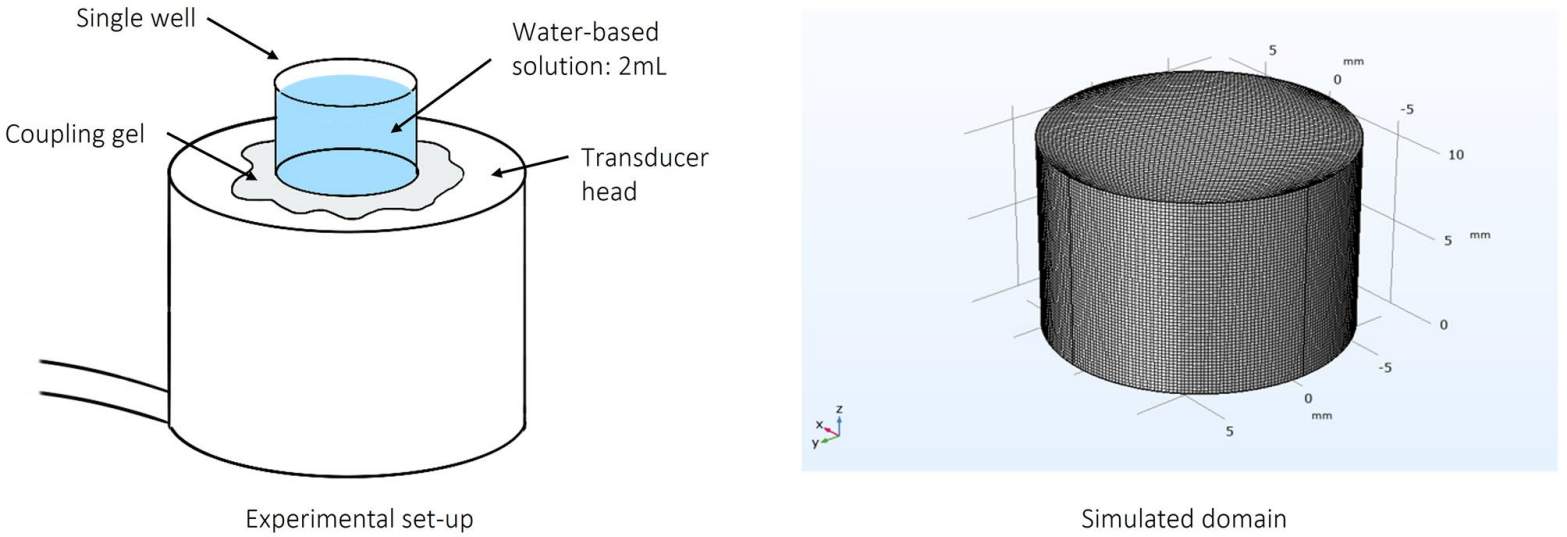
Scheme of the experimental set-up (left) and simulated domain (right), whose description and characteristic dimensions are reported in Table 1

The simulation parameters whose values have not already been expressed are listed in Table 1 and the geometrical features of the well containing the water volume as well as the position of the US transducer are depicted in Fig. 1.

**Table 1:** Some of the parameters used in the simulation. The ones related to water, polystyrene and air are referred to a temperature of 298.15 °K. The polystyrene attenuation coefficient was determined from the formula present in Reference (Takagi et al. 2007) and by using the values reported by some of us and by other authors (Spigarelli et al. 2020; Tiong et al. 2017; Lei et al. 2013). In the calculation of the air acoustic impedance, the speed of sound in the air and the air density have been taken respectively from the work of Dhiren M Joshi and Shih (Shih and Shih 2019; Joshi n.d.)

**Parameter**	**Value**	**Description**
$\mu$	0.890 [mPa·s]	Water dynamic viscosity (Snehota et al. 2020)
${\mu }_{B}$	2.485 [mPa·s]	Water bulk viscosity (Snehota et al. 2020)
$\rho$	998 [Kg·m^−3^]	Water density (Snehota et al. 2020)
α	0.108 [cm^−1^]	Polystyrene attenuation coefficient (Spigarelli et al. 2020), ^X^
Z_ps_	2.5·10^6^ [Pa·s·m^−1^]	Polysterene acoustic impedance (Snehota et al. 2020)
Z_air_	4.017·10^2^ [Pa·s·m^−1^]	Air acoustic impedance (Racca et al. 2020; Garino et al. 2019)
r	7.950 [mm]	Well basis radius
H	10.78 [mm]	Solution height
L_D, 0.30_	9.95 [nm]	Bottom wall displacement at I_input_ = 0.30 W/cm^2^
L_D, 0.45_	12.19 [nm]	Bottom wall displacement at I_input_ = 0.45 W/cm^2^
L_D, 0.60_	14.06 [nm]	Bottom wall displacement at I_input_ = 0.60 W/cm^2^

### Synthesis, functionalization and preliminary characterization of zinc oxide nanocrystals

ZnO nanocrystals (NCs) were synthesized through a hydrothermal microwave-assisted approach developed by Garino et al. (2019). More details are reported in the Supplementary Information. Then, the ZnO NCs were functionalized with amino-propyl groups using 3-aminopropyltrimethoxysilane to obtain ZnO-NH_2_ NCs, as detailed in the Supporting Information (S.I.) and in ref Garino et al. (2019).

The crystalline structure of the ZnO-NH_2_ NCs was analyzed by X-ray diffraction (XRD) with a Panalytical X’Pert diffractometer in θ − 2θ Bragg–Brentano configuration, having a radiation source of Cu-Kα, with λ = 1.54 Å, 40 kV and 30 mA. The sample was prepared by depositing and let drying some drops of the NCs colloidal suspension on a flat surface.

High Resolution Transmission Electron Microscopy (HRTEM) was performed on FEI Titan ST microscope, at acceleration voltage of 300 kV, equipped with a S-Twin objective lens, an ultra-bright field emission electron source (X-FEG) and a Gatan 2 k × 2 k CCD camera. The imaging was obtained by depositing a drop of the samples (NCs diluted in ethanol with a concentration of 100 μg/mL) on a holey carbon copper grid and dried overnight.

Finally, the hydrodynamic size and Z-potential value of ZnO-NH_2_ NCs in distilled water suspension was determined through the Dynamic Light Scattering (DLS) technique with a Zetasizer Nano ZS90 from Malvern.

### Experimental acoustic stimulation of solutions

Ultrasounds were generated by using the LipoZero G39 (GLOBUS) unfocused transducer. Each sample was composed of a water-based solution in a single well of a 24 well plate of polystyrene (PS, Thermo Scientific) and positioned on the Us transducer (Fig. 1). To ensure the contact between the sample and the transducer, a thin layer of coupling gel (ELvation Medical GmbH) was interposed. The experiments were performed by setting the following parameters: ultrasound frequency of 1 MHz, Duty Cycle (DC) of 100%, power densities (i.e. acoustic density generated by the transducer) of 0.3, 0.45 and 0.6 W/cm^2^ and sonication times of 5 s, 10 s, 15 s, 30 s, 1 min, 2 min.

Pressure inside the well was measured with Ago Hydrophone SN2195, the instrument was placed in the center of the well and the bd water solution was insonated at 1 MHz, 100% Duty Cycle (DC) and different input power densities. Temperature increase due to the US exposure was evaluated by a thermocouple, starting at standard temperature.

The ROS generation was analyzed in pure bidistilled (bd) water solutions (from Milli Q, Millipore-Merck) or in water-containing ${\mathrm {ZnO-NH}}_{2}$ NCs at 200 μg/mL concentration.

The amount of ROS was evaluated with two different detection techniques: the induction and successive analysis of sonochemiluminescence (SCL) from luminol solutions and the Electron Paramagnetic Resonance (EPR) Spectroscopy assisted by a spin-trapping technique, as detailed below.

### ROS evaluation with sonochemiluminescence method

As described in the work of McMurray et al. (1999), SCL is the emission of blue light (λ = 430 nm) by Luminol molecules in an alkaline solution caused by their interaction with hydroxyl radicals (OH·), generated by acoustic cavitation. This oxidative chemiluminescence of Luminol develops through a complex reaction pathway of the molecule, which undergoes many intermediate reaction steps before emitting the blue light. For a given concentration of luminol molecules in the solution, the light intensity emitted (and then detected) by SCL, denoted as ${I}_{SCL}$, is proportional to the amount of cavitation-generated hydroxyl radicals in water.

To ensure a proper activation of the SCL process, a solution composed of 80 mM luminol (97% by Sigma-Aldrich), 0.1 M sodium hydroxide (NaOH) and 0.5 mM hydrogen peroxide (${H}_{2}{O}_{2}$, from a stock solution of 0.02 M) in a total volume of 2 mL of bd water was placed in a well of the 24 well plate. Furthermore, in case of nanoparticles-assisted US study, ${\mathrm {ZnO-NH}}_{2}$ NCs were added leading to a final concentration of 200 μg/mL. The prepared solutions were treated within 4 h from their preparation.

**Fig. 2:**
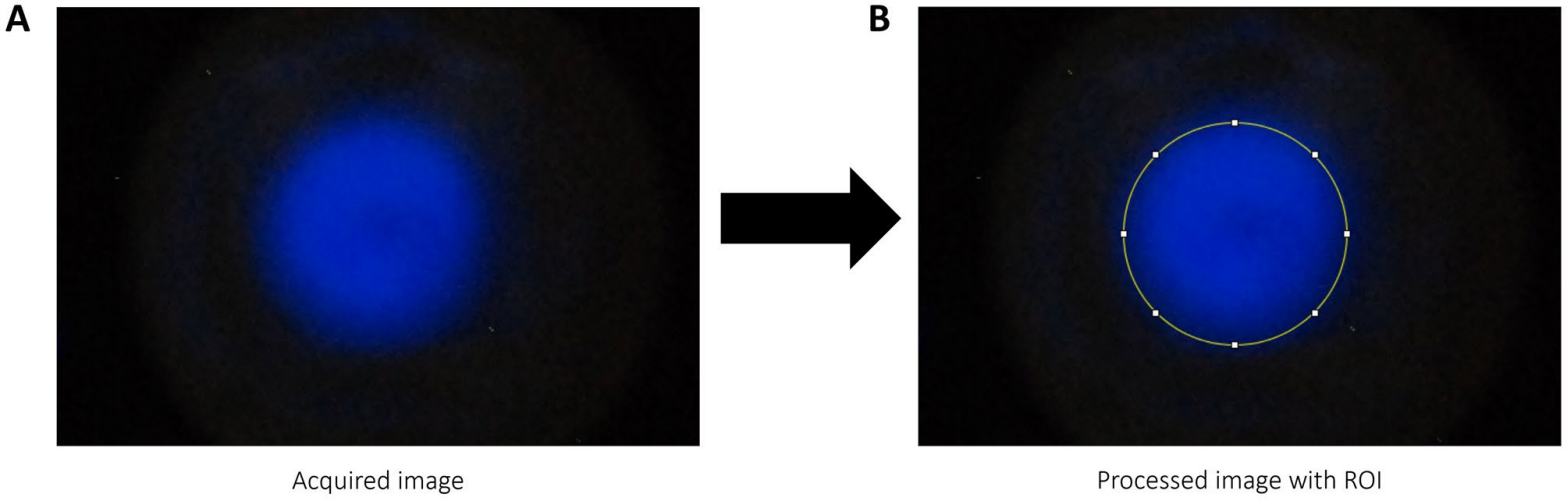
Acquired image from the 24-well plate and its processing. The image resulting from the sonication **A** is subjected to subtraction of the background image and the selection of a specific circular ROI **B**. These images refer to the particular sonication condition with US power = 0.6 W/cm^2^ and sonication time 3 min

The SCL intensity emitted by each solution during the sonication was detected by using a Nikon D80 digital camera, set with ISO = 1250, f-stop = f/4 and with an exposure time that, for the shorter sonication times (as 5 s, 10 s, 15 s) is at least of 30 s, while, for the longer sonication times (as 30 s, 1 min, 2 min), it is set equal to the sonication time. Thus, by fixing the camera on a tripod and positioning it right above the well (approximately at a height around 20 cm from the well), images of the blue colored solutions were acquired, showing a visible circle corresponding to the well shape (Fig. 2A). Following the work of Tiong et al. (2017), to avoid the acquisition of noisy light coming from the environment (due to the long exposure times) and remove the thermal noise inside the camera, the experiments were performed in a dark room and a frame image was taken in absence of sonication (background) before each experiment. After the experiments, the obtained images were analyzed and processed with the open-source software ImageJ: firstly, the background was subtracted to the acquired images in a pixel-by-pixel approach; then, the area of the images showing blue light pixels (Fig. 2A) was selected as Region Of Interest (ROI, Fig. 2B) and spatial average blue intensity was calculated over all the intensity values of the pixels inside the ROI. The processed images were set as 24-bit RGB color images and since Luminol emits blue light, only the intensity of the blue channel was computed.

### ROS evaluation by Electron Paramagnetic Resonance (EPR) Spectroscopy

A quantitative evaluation of ROS production by US-induced cavitation in a water-based solution was provided by means of the Electron Paramagnetic Resonance (EPR) Spectroscopy, performed with the EMXNano X-Band spectrometer (Bruker).

As previously reported (Vighetto et al. 2019), the formation of both hydroxyl and superoxide anion radicals was measured with 5,5-dimethyl-L-pyrroline- N-oxide (DMPO, Sigma), employed as spin-trap. In particular, 2 ml of bd water with 10 mM of DMPO were irradiated by US at different power and times in the well of a 24 well plate. In addition, 200 µg/mL of ZnO-NH_2_ NCs in 2 mL of bi-distilled water, together with 10 mM of DMPO were also tested.

Immediately after sonication, 50 µl was collected through a quartz microcapillary tube and analyzed by EPR. The obtained spectra were acquired with the following conditions: 3428 G for the center field, 60 s of sweep time, 60 as receiver gain, 2.00000 as g-factor and 10 as number of scans. For each analyzed sample, the resulting molar concentration of DMPO-OH molecules were extrapolated from the acquired spectrum with the *SpinFit* software; null molar concentration was considered for spectra not having any recognizable trend.

### Cells line and treatments

The human Burkitt Lymphoma cell line Daudi (ATCC^®^ CCL-213TM), as a suspension cell line, was cultured in RPMI-1640 Medium (ATCC30-2001) supplemented with 10% of heath inactivated fetal bovine serum (ATCC-302020), 100 units/mL penicillin and 100 μg/mL streptomycin (Sigma-Aldrich, Schnelldorf, Germany) and maintained at 37 °C under a 5% CO_2_ atmosphere. To evaluate the effects of ultrasound irradiation on this hematological cancer cell line, samples containing 2 × 10^5^ cell/mL were plated into 24 well plate (Nunc) with 2 mL per well, treated with different conditions of ultrasounds and promptly seeded in a 96 well plate (Greiner Bio-One). Each 96 well contained 100 μl per well of sample solution, in three replicates for viability assay. After 24 h indeed, 10 µl/well of cell proliferation reagent WST-1 (Roche) was added and after additional 4 h of incubation, the formazan absorbance was detected at 450 nm by the Multiskan Go microplate spectrophotometer (Thermo Fisher Scientific Waltham, MA, USA) using a 620 nm reference.

The effects of ultrasound and the further synergistic action of US and amino-functionalized ZnO nanocrystals were tested on another cancer cell line. i.e. cervical adenocarcinoma KB cell line (ATCC CCL17TM), which grows in adhesion as a 2D monolayer. KB cells were grown in Eagle’s Minimum Essential Medium (EMEM, Sigma) supplemented with 10% heat inactivated fetal bovine serum (FBS, Sigma), 100 units/mL penicillin and 100 µg/mL streptomycin (Sigma), and maintained as reported for Daudi cell line (Racca et al. 2020; Vighetto et al. 2021).

Before treating the KB cancer cells with US, cells were trypsinized, counted, and 5 × 10^5^ cells were plated in 1 mL of complete medium/well into 24 well plates (Nunc). In this way, KB cell were in suspension in cell culture media and thus exposed to ultrasound irradiation. Different experimental groups were considered: untreated cells, cells treated with US, and cells pre-treated for 24 h with ZnO-NH_2_ NCs with or without the subsequent US treatment. In the case of cells pretreated with ZnO-NH_2_ NCs, cells were preincubated with 10 µl/mL ZnO-NH_2_ NCs for 24 h then trypsinized and counted following the same protocol as before. After the treatment, cells were promptly seeded in a 96 well plate (Greiner Bio-One), i.e. at 2 × 10^3^ cells in 100 μl medium per well in replicates, for the viability assay. After 24 h from the treatment, proliferation activity of KB cells was measured by WST-1 assay, as previously reported for Daudi cells.

All cells were treated with LipoZero ultrasound transducer, with US frequency equal to 1 MHz, 100% of Duty Cycle, and different condition of power densities (0.3, 0.45 and 0.6 W/cm^2^) and exposure times, as detailed above.

All the reported results are expressed as percentage of cell viability with respect to untreated cells, assessed as 100% viable. Data are shown as mean ± standard deviation.

## Results and discussion

### Acoustic field simulations

**Fig. 3:**
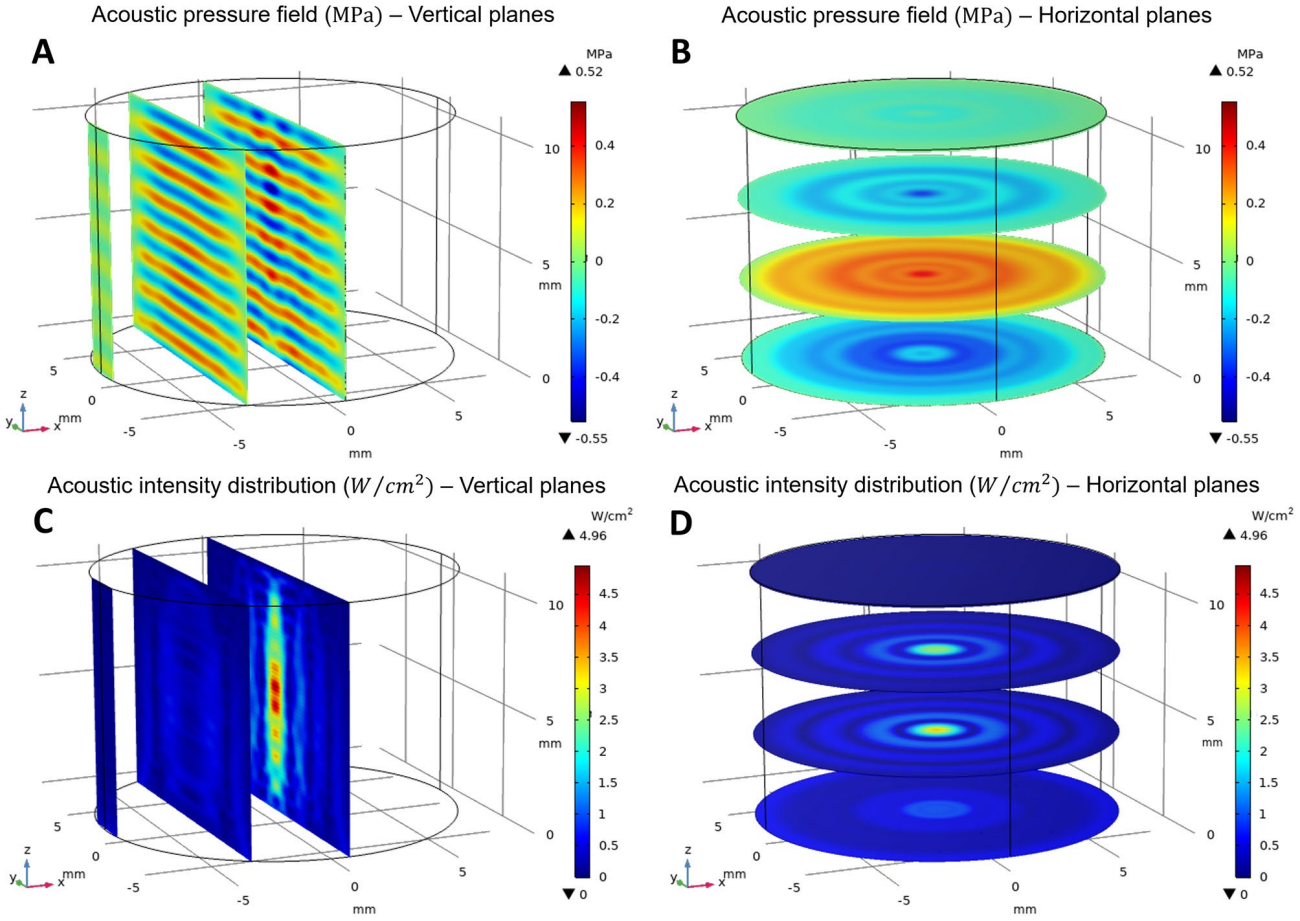
Acoustic pressure field (top panels) and acoustic density distributions (bottom panels) within the well simulated with COMSOL^®^ Multiphysics for I_input_ = 0.45 W/cm^2^, along vertical (**A** and **C**) and horizontal (**B** and **D**) planes of the sample well containing water. The darkest red and blue colors in panels A and B indicate the maximum and the minimum values, respectively, that the acoustic pressure can assume, equal to + 0.52 MPa and—0.55 MPa. The darkest blue and red colors of the acoustic density (panels **C** and **D**) correspond to 0 W/cm^2^ and 4.96 W/cm^2^, respectively

The simulations allowed to model the acoustic pressure field and the acoustic density distribution inside the well due to the propagation of the US wave through the solution. In Fig. 3 both entities are computed at I_input_ = 0.45 W/cm^2^ of the US transducer input density (i.e. acoustic density generated by the transducer), while other power densities are considered in the S.I.

The acoustic pressure field and the acoustic density distribution inside the well are visualized along three vertical planes (Y–Z planes, Fig. 3A and C), selected at x = 0 (well central section), x = 3.98 mm (half of the well basis radius along the X axis) and x = 7.9499 (0.0001 mm from the well wall along the X axis). These vertical planes allowed to study the US behaviour along the wave propagation direction (i.e. the vertical axis, Z) and when moving radially (X and Y axes). A second set of planes was selected horizontally (X–Y planes, Fig. 3B and D), chosen at z = 0 (well bottom surface), z = 2.5 mm (one quarter of the well height), z = 7.5 mm (three quarters of the well height) and z = 10.7 mm (well top surface).

The acoustic pressure field on the vertical planes (Fig. 3A) highlights the alternation of compression and rarefaction cycles along the Z axis, according to the propagation direction of US. This trend is sinusoidal, characterized by highly varying and pronounced peaks around the vertical central axis, in particular at the well center, and then tend to decrease and to assume a more regular and homogeneous shape when moving radially towards the walls. Also from the acoustic pressure field on the horizontal planes (Fig. 3B), it can be observed that the peaks around the central vertical axis gradually decrease along the radial direction, following an oscillatory trend of symmetrical pressure phases. This behavior may be caused by the scattering of the propagating US wave inside the fluid and by its interaction (transmission and reflection phenomena) with the cylindrical lateral surfaces of polystyrene walls and with the air at the top surface, generating specific interference effects. In fact, Fig. 3A demonstrates that the region around the central vertical axis appears to be subjected to strong constructive and destructive interference effects, originating diversified local maxima and minima pressure peaks which decrease along the radial direction. Moreover, it can be supposed that the particular axis-symmetric geometry of the well favors the formation of a symmetric and tapered focus along the central vertical axis, composed by the highest pressure amplitude values.

Further information about the behavior of the US field inside the well can be gained from the acoustic density distribution on the vertical (Fig. 3C) and horizontal planes (Fig. 3D): as previously noticed, a symmetric and tapered focus along the central vertical axis is clearly visible, showing the highest acoustic density values (at about 4.5 W/cm^2^) around the center of the well. From this region, the intensity slowly decreases moving towards the top and bottom surfaces, while it sharply reduces radially. Moreover, interference effects can be distinctly observed, represented by a pattern of concentric fringes, whose variations are strong around the vertical central axis and attenuate along the radial direction.

The same trends of the acoustic pressure field and the acoustic density distribution on the vertical and the horizontal planes were obtained also for I_input_ equal to 0.60 W/cm^2^ and 0.30 W/cm^2^ (reported in S.I. Figs. S.1 and S.2), even if they were characterized by different values, as expected by the different US input powers densities used and thus of the different bottom wall displacements of the well.

Finally, a comprehensive analysis was carried out by computing the average acoustic pressure amplitude and acoustic density on each of the three vertical planes, for all the three US input power densities (0.3, 0.45 and 0.6 W/cm^2^ in Table 2). The obtained data show that for each US power density in input, both obtained acoustic pressure and density decrease along the radial direction, in agreement with the previous color maps of Fig. 3. A higher reduction is achieved by approaching the well lateral surface. At the same time, for each vertical plane, the average acoustic pressure amplitude and acoustic intensity increase proportionally to the transducer power density, as expected. It is worth to note that the average acoustic density calculated in the central plane of the well is higher than the power density of the transducer. This local value provides the highest contribution to the average density over the whole domain, However, it is counterbalanced by the much lower acoustic densities calculated at the well boundaries. As a result, the average density over the whole solution volume is actually lower than the transducer input power one. This outcome can be attributed both to the viscous losses of the fluid (which are taken into account in the used *Viscous Fluid* model) and to the acoustic pressure and density trends described above.

**Table 2:** Average acoustic density and acoustic pressure amplitude computed on the three considered vertical planes and at different US power densities in input from the transducer

**Transducer input power density [W/cm**^**2**^**]**	**0.3**	**0.45**	**0.6**
Bottom wall displacement [nm]	9.95	12.19	14.06
Density average central [W/cm^2^]	0.414	0.621	0.826
Density average r/2 [W/cm^2^]	0.193	0.290	0.386
Density average wall [W/cm^2^]	0.070	0.105	0.140
Density average whole solution [W/cm^2^]	0.236	0.355	0.472
Pressure ampl average central [MPa]	0.152	0.187	0.215
Pressure ampl average r/2 [MPa]	0.125	0.154	0.178
Pressure ampl average wall [MPa]	0.043	0.052	0.061

### Experimental measurements

With the aim to validate the acoustic pressure and density simulations with experimental data, the central average acoustic pressure was evaluated in the center of the 24-well at each US input power densities, as used for the mathematical simulations, by hydrophone measurements. The collected data are reported in the S.I, Fig. 2.4 showing that the same values of acoustic pressure inside the well are achieved with both simulation and experimental tests, and thus perfectly validating the output results and the correctness of the used mathematical model.

Furthermore, the ROS production was investigated in the well volume under US irradiation at the selected input conditions and measured through the SCL and EPR methods (Vighetto et al. 2019; Podbevšek et al. 2022). Initially, the ROS measurements were conducted in pure water, as previously considered for the simulations, and afterwards the same US input power densities and times were applied to living cancer cells, i.e. KB and Daudi cell lines, both in suspension in their culture media. The effect of ROS in living cell is multifaced, due to their role in the cell signaling system (Fu et al. 2014). An imbalance, i.e. an overproduction, of ROS levels in cells can result in the activation of apoptotic pathways, which in turns lead to cell deaths (Dąbrowski 2017). On the other hand, the mechanical stress generated by acoustic cavitation (Mathias et al. 1991), responsible for ROS production, and occurring close to cells membrane, can damage the integrity of the cell, being responsible for necrotic cell death. Therefore, it is very interesting to complete the above-reported simulation and the experimental ROS measurements with data of cell viability in the same US exposure conditions. On the other way round, the evaluation of cell viability can be a meaningful tool to indirectly assess the absence or the presence of abnormal ROS production in an US irradiated cell culture solution, further supporting the EPR and SCL measurements and the simulation results.

In a second set of experiments, the role played by ZnO-NH_2_ NCs dispersed in water was evaluated in terms of ROS generation and thus on the possible amplification of US effects. Ultimately, the analysis of the KB cancer cells viability after 24 h of incubation with NCs and consequent US treatment was examined, trying to propose a correlation among ROS generation and the cell viability, also depending on the presence of nanoparticles. It is worth to mention that the selected US power densities used to run both simulations and experiments are dictated mainly by the cell viability: actually, preliminary experiments showed that US input densities higher than 0.6 W/cm^2^ relied on a rapid increase of the temperature of the water media and on a high percentage of cell death, even without the presence of NCs, as reported in S.I., Fig. S.5. Since these effects prevented to evaluate the non-thermal effects of US on the cell fate, higher US powers than 0.6 W/cm^2^ were not considered in the present study.

#### Measurement of acoustic cavitation in pure water and on living cancer cells

**Fig. 4:**
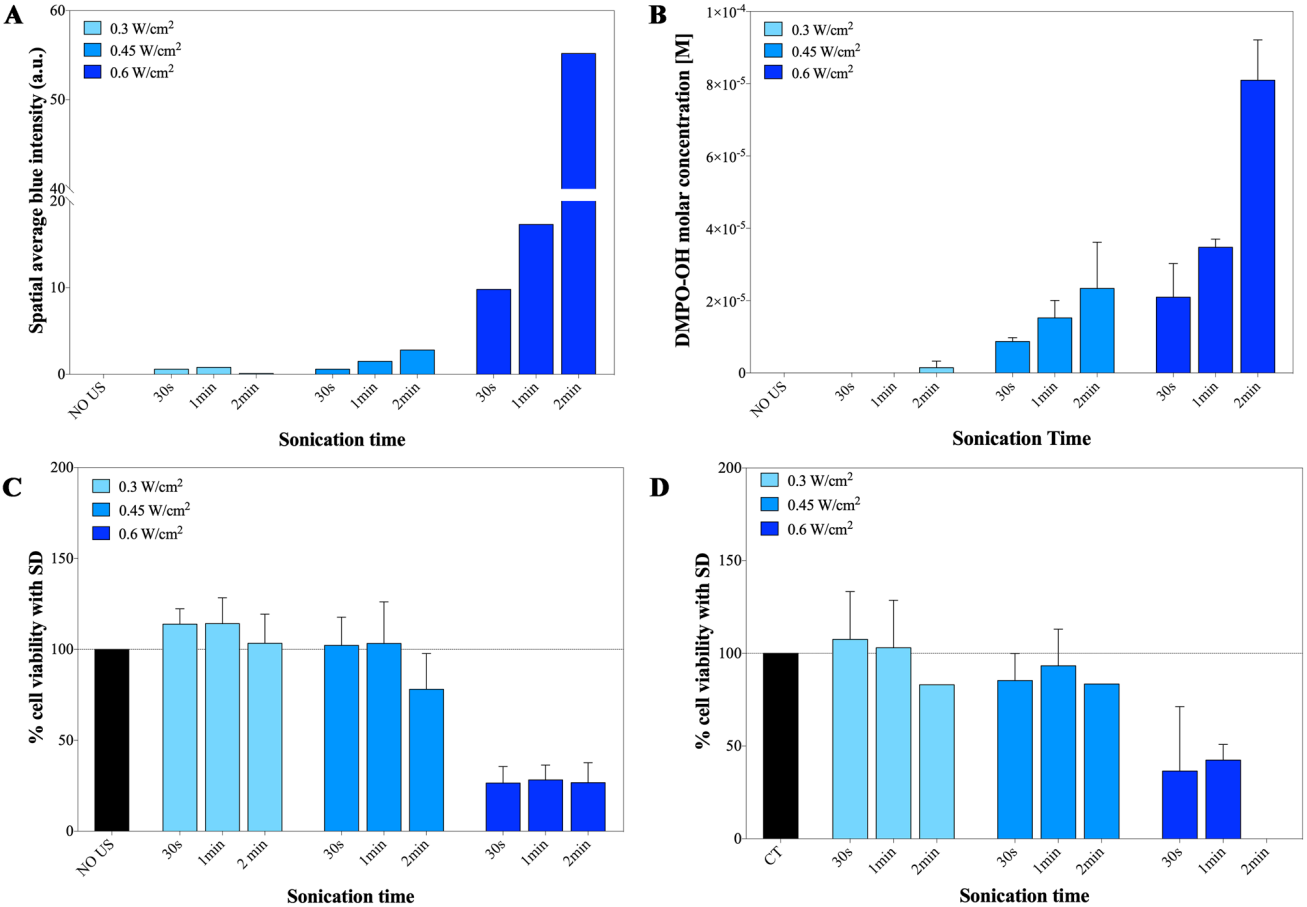
The generation of ROS in bd water volumes and the possible effects on living cell cultures: (**A**) Spatial average blue intensity of luminol due to sonochemiluminescence (SCL); (**B**) Molar concentration of DMPO-OH spin adducts obtained during EPR measurements; (**C**) KB cancer cell viability and (**D**) Daudi cell viability after 24 h from US irradiation. In these experiments, UT means untreated cells, and the presence of ZnO-NH_2_ NCs is not considered

Figure 4 compares the production of ROS, evaluated by SCL of Luminol and EPR measurements of DMPO-OH molar concentrations, with the cell viability essays on two different cancer cell lines (KB cells, a cervical adenocarcinoma and Daudi cells, a Burkitt lymphoma) at different US input power densities and irradiation times. Studying the effect of US on two so different cancer cell lines, allow us to test also the biological variability, enabling us to verify the hypothesis of a potential therapeutic system.

Both the spatial average SCL blue intensity detected from the well (Fig. 4A) and the molar concentration of DMPO-OH spin adducts found through EPR measurements (Fig. 4B) globally show an increasing trend proportional to longer sonication times and higher US power densities in input. Considering firstly the lowest US power density (0.3 W/cm^2^), the related SCL intensity assumes negligible values for all the sonication times, in agreement with the null molar concentration of DMPO-OH obtained by EPR. This result corresponds to a negligible production of ROS during sonication. At the same power density, a high cells viability is obtained for both cell lines. In particular, at the lowest applied US power of 0.3 W/cm^2^, KB cells are not affected by the US treatment (Fig. 4C), even for the longest sonication times (up to 2 min). Daudi cells also report a viability close to 100% for 30 s and 1 min, and a slight decrease of their metabolic activity is exhibited for 2 min sonication condition (Fig. 4D). The unvaried cell viability for almost all the tested conditions supports the EPR and SCL measurements in which ROS production is negligible.

Concerning the results obtained by applying the intermediate US power, 0.45 W/cm^2^, a negligible Luminol intensity is observed for 30 s sonication time (Fig. 4A), while it gradually increases from 1 to 2 min. In particular, at 0.45 W/cm^2^, a treatment of 1 min represents the shortest sonication time able to produce a detectable SCL blue light, even if its value is relatively very low. This result is consistent with the EPR results (Fig. 4B), showing a similar increasing trend of ROS production as the sonication time increases, i.e. from 30 s to 1 min and to 2 min of sonication time.

Concerning the cells viability, the first signs of modest cytotoxicity are obtained at 0.45 W/cm^2^ in KB cells at 2 min of sonication (around 78% cell viability in Fig. 4C), while are already visible at the shortest sonication time of 30 s in Daudi cell line (Fig. 4D). We hypothesize that the applied power density of 0.45 W/cm^2^ may impact on the cell viability, even slightly, while the dose of this treatment, which depends also on the application time, may vary depending on the cell type. More in general, the cell viability trend (Fig. 4C and D) appears to be globally lower at 0.45 W/cm^2^ than at 0.3 W/cm^2^ power density, and seems to be in line with the higher ROS production, increasingly proportional to the applied US power densities and times.

A remarkable behavior is presented by the measurements at 0.60 W/cm^2^ of applied US power density. Both the SCL intensity histogram (dark blue bars in Fig. 4A) and the molar concentration of DMPO-OH adducts (dark blue bars in Fig. 4B) show non-negligible and increasing values of ROS production as a function of the treatment time. This behavior is reflected by the average cell viability values below 30% (see dark blue bars in Fig. 4C and D) in both cancer cell lines, indicating an evident cytotoxic effect of 0.60 W/cm^2^ power density at any sonication time. In particular, the recorded cell viability of Daudi cell line is null at the highest tested time, i.e. 2 min.

Considering that both the cell lines are dispersed homogeneously inside the solution volume, it can be hypothesized that they are continuously moved by the convective forces generated by US during the irradiation time. It can be supposed that during the treatment, the majority of cells are exposed one or more times to the strongest sonication activities occurring in the central region of the well, as highlighted by the simulation results. Actually, the simulation revealed the presence of a central tapered region in the well with the highest values of acoustic pressure and intensity. From the experimental data of SCL intensity and DMPO-OH molar concentration, a consistent ROS production can be also noticed. All the above considerations can be used to possibly motivate the reduced cell viability recorded at the highest US power density used, i.e. 0.6 W/cm^2^.

#### Measurements of acoustic cavitation mediated by ZnO-NH_2_ NCs

The previous measurements allowed to set a base of understanding about the possible interaction among acoustic irradiation and living cell systems. Further comprehension of this mechanism can be done by challenging the overall system with the addition of inorganic metal oxide nanoparticles in the water solution, providing a direct evidence of the effect of solid-state inorganic nanoparticles compared to the pure water solutions. Furthermore, the ZnO-NH_2_ NCs were exploited in the cell culture media, allowing their internalization in KB cancer cells and providing a base of understanding in synergy with US irradiation. In these experiments, the lowest US powers, equal to 0.3 and 0.45 W/cm^2^, were applied since they have per se a non-toxic behavior towards cells, as demonstrated in Fig. 4C. For similar reasons, the ZnO-NH_2_ NCs concentration of 10 µg/mL was incubated with the KB living cancer cells, as it resulted from a previous study (Racca et al. 2020) to do not cause any cytotoxicity to the cells (in absence of US), and to provide a sufficiently high amount of NCs internalized into the cells (Racca et al. 2020). However, for the sake of completeness, also the results associated to an input power of 0.60 W/cm^2^ are reported in this study.

**Fig. 5:**
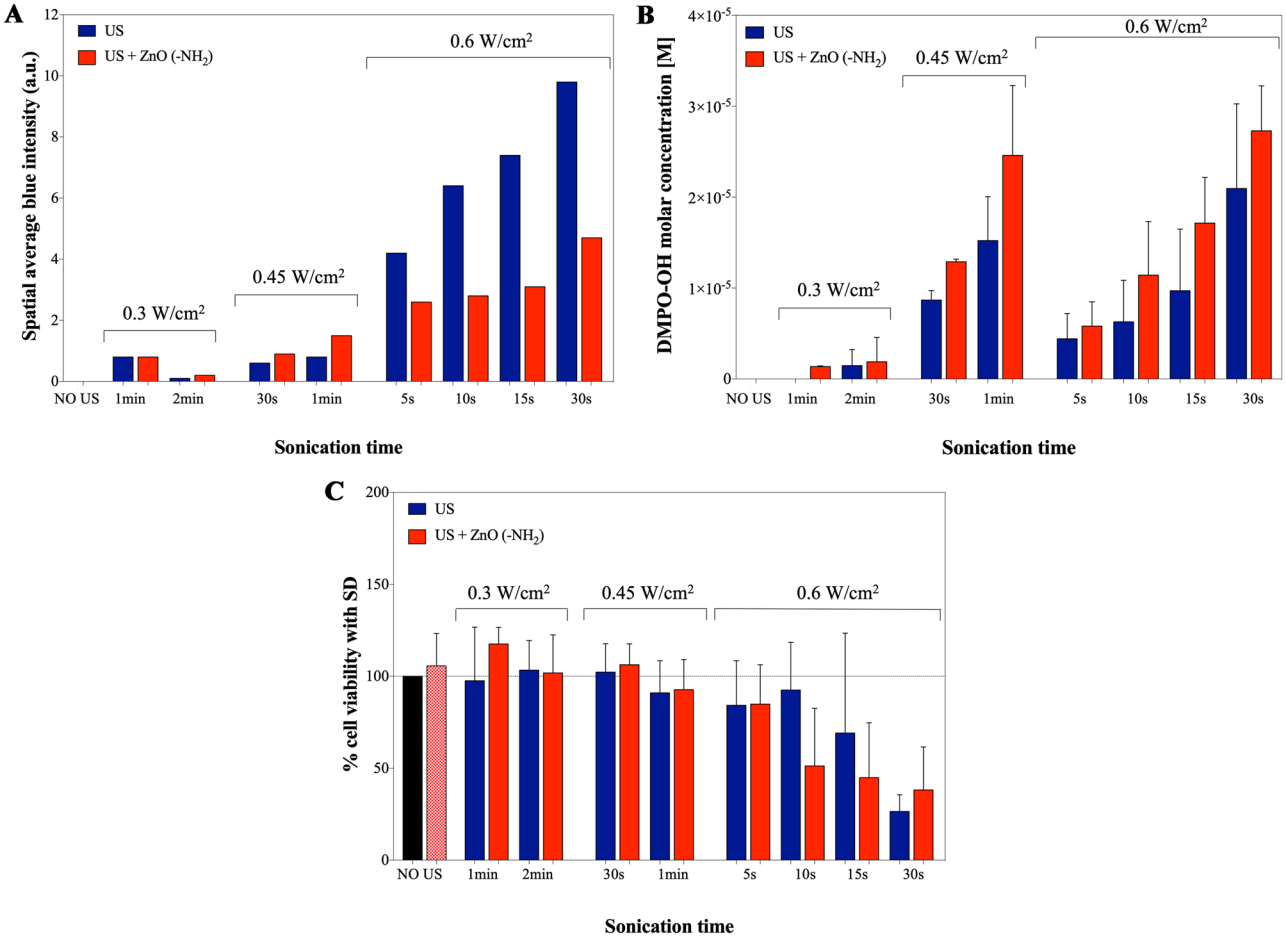
Overview of the ROS generation and related cell viability in a system comprising inorganic metal oxide nanoparticles (ZnO-NH_2_ NCs) and ultrasound irradiation in a well from a 24-multiwell plate. (**A**) Luminol blue intensity from SCL measurements with 200 μg/ml of ZnO-NH_2_ NCs; (**B**) DMPO-OH adduct concentration measurements from EPR spectroscopy to evaluate ROS production (with 200 μg/ml of ZnO-NH_2_ NCs), and (**C**) KB cancer cell viability according to different US power densities and sonication times (with 24 h preincubation of cell with 10 μg/ml of ZnO-NH_2_ NCs); black bar and red dotted bar correspond respectively to untreated KB cell and cells incubated with NCs without US exposure

Figure 5 shows an overview among the effect of US irradiation combined with the presence of NCs leading to the generation of ROS in water-based media (red bars), and a possible comparison with the cell viability, by analyzing the influence of different sonication times and input power densities. Control measurements in the absence of ZnO-NH_2_ NCs (blue bars) are also inserted for reference and refer to experiments already reported in Fig. 4.

An initial consideration can be done in absence of US irradiation, where the sole presence of ZnO-NH_2_ NCs is not sufficient for generating ROS, thus neither blue light emission in SCL (Fig. 5A), nor DMPO-OH adducts in EPR spectroscopy (Fig. 5B) are measured. These results are consistent with high cell viability of the NCs-incubated cells in absence of US (red dotted bar in Fig. 5C) very similar to the untreated cell control (black bar).

SCL measurements at 0.3 W/cm^2^ and 0.45 W/cm^2^ (Fig. 5A) show a slight increased spatial average blue intensity in presence of ZnO-NH_2_ NCs, in accordance with the augmented amount of free radicals detected, i.e. EPR measurements (Fig. 5B), when NCs are in solution. A different behavior is displaced in SCL at 0.6 W/cm^2^. The presence of NCs reduces the detected light compared to pure water under identical US irradiation conditions. As reported in literature (Vighetto et al. 2022; Özgür et al. 2005), ZnO NCs are characterized by an absorption spectrum (Fig. S-3) which partially overlap with blue light emission by SCL. It is possible to state that for 0.6 W/cm^2^, even though there is an increased ROS production with NCs (Fig. 5B) attributable to an increased inertial cavitation, the absorption by ZnO NCs of the photon produced by SCL become a dominant phenomenon and it is responsible for the diminished blue signal detected due to the close proximity between the NC and point of photon generation (Vighetto et al. 2022; Kwan et al. 2015; Suslick and Flannigan 2008).

EPR measurements (Fig. 5B) show a monotonic increase of DMPO-OH concentration values in presence of ZnO NCs(red bars in Fig. B) with respect to the values from pure water at the same conditions (blue bars in Fig. 5B). Therefore, a clear trend of the ROS generation as function of the sonication time and the US input power density can be observed and is enhanced by ZnO-NH_2_ nanocrystals. The trend of radical production, visible for all the power densities tested, suggests a constructive interaction between US and NCs, which possibly facilitated inertial cavitation in water, responsible for ROS production, and lowers the cavitation threshold, as also previously reported (Vighetto et al. 2019; Ancona et al. 2020).

In accordance with the results presented above, the US powers at lowest input densities, i.e. 0.3 and 0.45 W/cm^2^, did not impact significantly on the cancer cell viability in either presence or absence of NCs. While the presence of ZnO NCs resulted to increase the ROS production, this effect was not sufficient enough to induce cytotoxicity. Specifically, nor the used US power densities and times, neither the ZnO-NH_2_ NCs co-incubation, were sufficient to generate an irreversible damage to cells, (including the generated ROS or any mechanical effects) to cause a consistent cell death. Cell viability actually remained well above 90%.

Different considerations can be done for the highest US input power density, 0.6 W/cm^2^: at increasing irradiation times from few seconds (5–10 s) up to 30 s, a negative effect on cell viability is recorded, decreasing the cell viability down to 25–30% at 0.6 W/cm^2^ for 30 s, both in presence and absence of ZnO-NH_2_ NCs. The contribution of the NCs remains however unclear and the responsibility of the observed cell death mechanism can be mainly reconducted to the US input power density used and to a specific time of US application. Both can lead to ROS production, however, as mentioned above, ROS are not the only responsible for cell death, as far as also mechanical damages can be obtained and are here not measured.

The obtained finding however highlights the adequacy of the mathematical modelling and of both SCL and EPR experiments in pure water, all useful to quantify a certain “dose” of US given to a system and to hypothetically predict the possible biological consequences. In particular we highlight that the measurement of the luminol sonochemiluminescence, although not quantitative, may represent a useful qualitative method to rapidly and directly estimate the presence of ROS produced in an US irradiated solution and screen out conditions which could be applied to living cancer cell aiming at killing them. Clearly, the final proof of the identification and quantification of the produced ROS has to be achieved with a more robust technique, like the EPR spectroscopy, mandatory in presence of optically responsive materials, which is however much more time consuming and laborious, and the biological results proofed by robust cell viability tests.

## Conclusion

In the present study we aimed to deepen the physical knowledge low-intensity ultrasound irradiating a simple water volume, producing reactive oxygen species and further evaluate a possible therapeutic activity against cancer cell.

We explored different US input power densities at low frequency (1 MHz) and we set a mathematical model able to predict the acoustic pressure field and acoustic density distribution in the analyzed water volume. Despite the used US were non-focused, we observed the formation of a symmetric and tapered focus along the central vertical axis of the water sample volume, having the highest pressure amplitude values and acoustic densities.

We then validated the simulated data with experimental measurements, detecting the reactive oxygen species generation with two different techniques, sonochemiluminescence and electron paramagnetic resonance spectroscopy. Both methods indicated a negligible value of produced ROS once US input power density are low enough, i.e. at 0.3 W/cm^2^, for any of the application time studied. An increasing trend of produced ROS is consistently observed once US power densities in input are increased and at increasing values of irradiation times. A parallel evaluation of two different cancer cell line viability was also performed. High cell viability was indeed accounted at the lowest US power densities in input, while a decrease in cell viability was recorded at higher power densities and increasing application times. In these conditions, the 0.6 W/cm^2^ US power density resulted to produce the highest amount of ROS and the strongest cytotoxic effect, especially for the longest treatment time of 2 min.

To further challenge the use of US in combination with nanoparticles, towards the direction of US-assisted nanomedicines, the interplay among US and amine-functionalized ZnO NCs was analyzed for any of the US power densities and times considered. Interestingly, the amount of ROS generated in presence of NCs is higher than the respective amount produced in pure water. This result suggested a constructive interaction between US and NC, which possibly facilitated inertial cavitation in water, responsible for ROS production, and lowers the cavitation threshold. The cell viability measurements were conducted first internalizing the nanoparticles for 24 h in KB adenocarcinoma cell line and then exposing the cells to US. Since the ZnO-NH_2_ NCs were adopted with cells in a sub-toxic concentration, a very good cell viability was observed both in absence of US irradiation and at the lowest US input power density, 0.3 W/cm^2^, irrespectively to the applied sonication time. In contrast at increasing US input densities of 0.45 and 0.6 W/cm^2^ and related increasing application times, more evident signs of cytotoxicity were observed, however the presence of ZnO-NH_2_ NCs did not give a clear contribution improving the cell cytotoxicity. These results evidence that the interplay among US and NPs in biological living system is more complex than in pure water and more conditions have to be explored prior to achieve a univocal answer. In our contest, it seems that US power densities and application times on a biological system plays the major role in dictating the cell viability.

As a whole, the obtained results fairly show that mathematical simulation of an acoustically treated water system can be also experimentally validated with simple measurements, like ROS production. In particular, the qualitative SCL measurements are confirmed in pure water by more robust and quantitative EPR technique and allow to estimate the amount of produced ROS in the system. In a further step, the amount of produced ROS can fairly correlate to the cancer cell viability measurements.

Particular attention should be paid when optically responsive materials, as ZnO NCs, are involved in the studied system. The interactions between all the elements become complex, involving multiple physical and chemical processes, and the mathematical model here proposed should be implemented. Further work will be done in this direction, moving towards a mathematical model able to predict the outcome of a complex system in which a large number of agents plays different roles, anticipating the biological behaviors of different cell lines.

## Supplementary Information

Below is the link to the electronic supplementary material.

Supplementary file1 (DOCX 2620 KB)
