# Supplementary material for: A comparative analysis of low intensity ultrasound effects on living cells: from simulation to experiments
Source: Biomed Microdevices. 2022 Oct 24;24(4):35. doi: 10.1007/s10544-022-00635-x (PMC9592626; doi:10.1007/s10544-022-00635-x)
Supplement: Supplementary file 1 — Supplementary file1 (DOCX 2620 KB) [file 10544_2022_635_MOESM1_ESM.docx]

**Supporting information**

A comparative analysis of low intensity ultrasound effects on living cells: from simulation to experiments

Giulia Tamboia^1^, Michele Campanini^1^, Veronica Vighetto^1^, Luisa Racca^1^, Luca Spigarelli^1^, Giancarlo Canavese^1^, Valentina Cauda^1*^

^1^ Politecnico di Torino, Department of Applied Science and Technology, Corso Duca degli Abruzzi 24, 10129 Torino, Italy

**Synthesis, functionalization and preliminary characterization of ZnO-NH_2_ NCs**

Firstly, zinc acetate di-hydrate (Zn(CH_3_COO)_2_ · 2H_2_O Puriss. p.a., ACS Reagent, ≥ 99.0% Fluka), chosen as zinc precursor and consisting in a fine white powder kept under vacuum condition since it easily reacts with water, is weighted and dissolved in methanol to obtain a concentration 0.09 M. Then, this opaque white solution is directly stirred in the Teflon reactor vessel, with the addition of double-distilled water, leading to a completely transparent solution. Also a potassium hydroxide solution (KOH ≥85% pellets, Sigma-Aldrich) composed of KOH dissolved in methanol with a concentration 0.02 M, is introduced inside the vessel, whose temperature and pressure are constantly controlled with specific probes. The final solution is then inserted in a microwave oven for 30 min at 60◦C.The reaction leading to the formation of ZnO nanocrystals relies on the hydrolysis of the zinc precursor induced by the presence of the hydroxide inside the solution.
After that, the solution is cooled down at room temperature and centrifuged for 10 min at 3500 G so that the NPs precipitate and it is possible to remove the solvent and the unreacted compounds. This procedure is carried on for two times to completely replace the solvent with ethanol (Sigma-Aldrich, 99%). Finally, ZnO nanocrystals are dispersed homogeneously through US sonication.

The desired quantity of ZnO nanocrystals dispersed in ethanol are drawn and inserted in a round-bottom glass flask, heated up to 80 °C and subjected to stirring and nitrogen gas flow. To provide the NCs functionalization, 3-aminopropyltrimethoxysilane (H_2_N(CH_2_)_3_Si(OCH_3_)_3_ APTMS 97%, Sigma Aldrich) is introduced to the solution at 10 mol% of the total ZnO amount. After 6 hours, two washing steps are performed through centrifugation (10 min, 10000 RCF) to remove all the unreacted APTMS and replacing it with ethanol.

Acoustic field simulations of 0.30 and 0.60 W/cm^2^


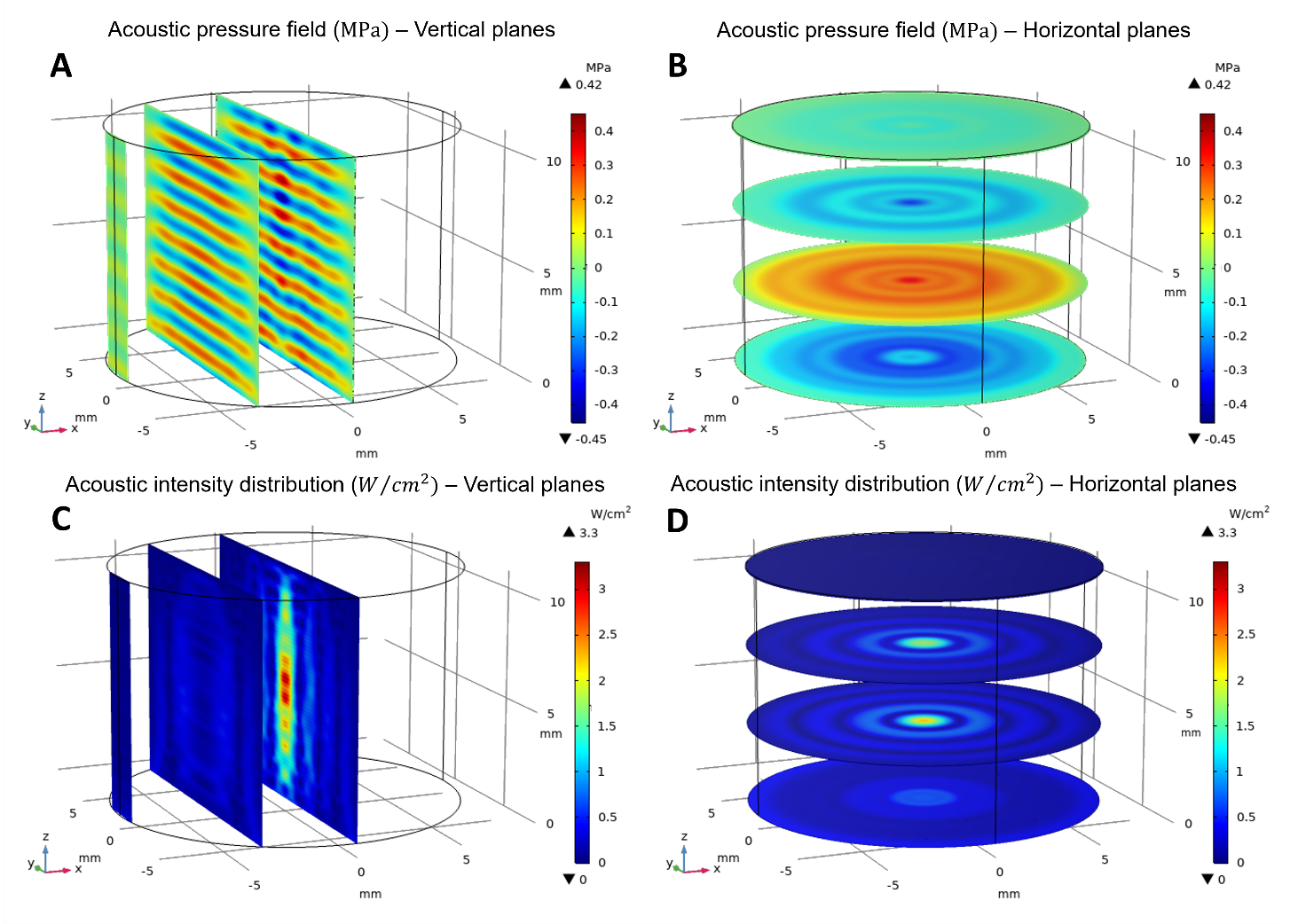


**Figure S-1.** Pressure (top panels) and intensity distributions (bottom panes) within the well simulated with COMSOL Multiphysics, along vertical (A and C) and horizontal (B and D) sections of the sample well filled with water. The US power in input was set to 0.30 W/cm^2^. The darkest red and blue colours in panels A and B indicate the maximum and the minimum values, respectively, that the pressure can assume. The darkest blue and red colours of the acoustic intensity (panels C and D) correspond to 0 W/cm^2^ and 3.3 W/cm^2^, respectively.


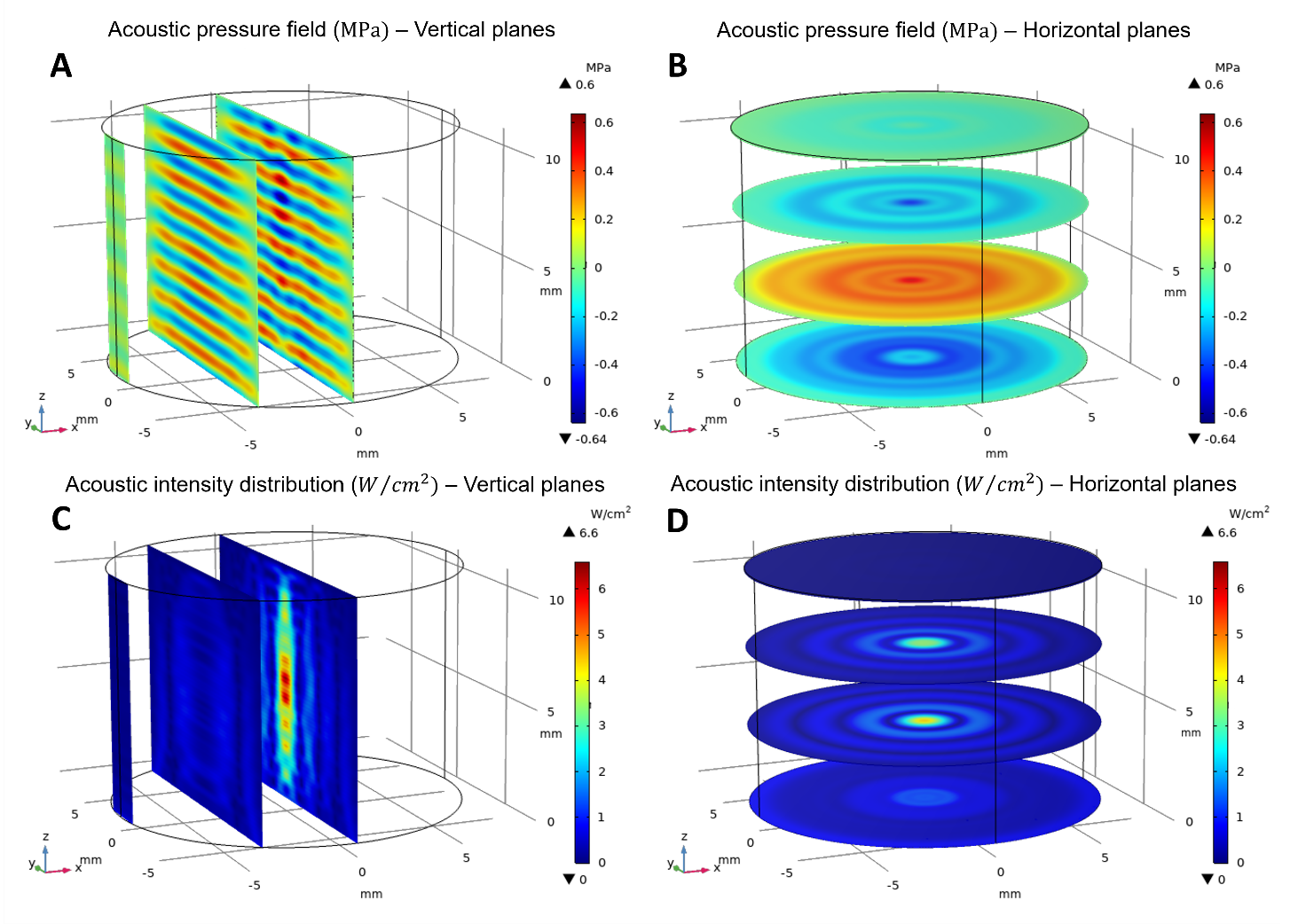


**Figure S-2.** Pressure (top panels) and intensity distributions (bottom panes) within the well simulated with COMSOL Multiphysics, along vertical (A and C) and horizontal (B and D) sections of the sample well filled with water. The US power in input was set to 0.60 W/cm^2^. The darkest red and blue colours in panels A and B indicate the maximum and the minimum values, respectively, that the pressure can assume. The darkest blue and red colours of the acoustic intensity (panels C and D) correspond to 0 W/cm^2^ and 6.6 W/cm^2^, respectively.

Absorbance spectrum of ZnO NCs


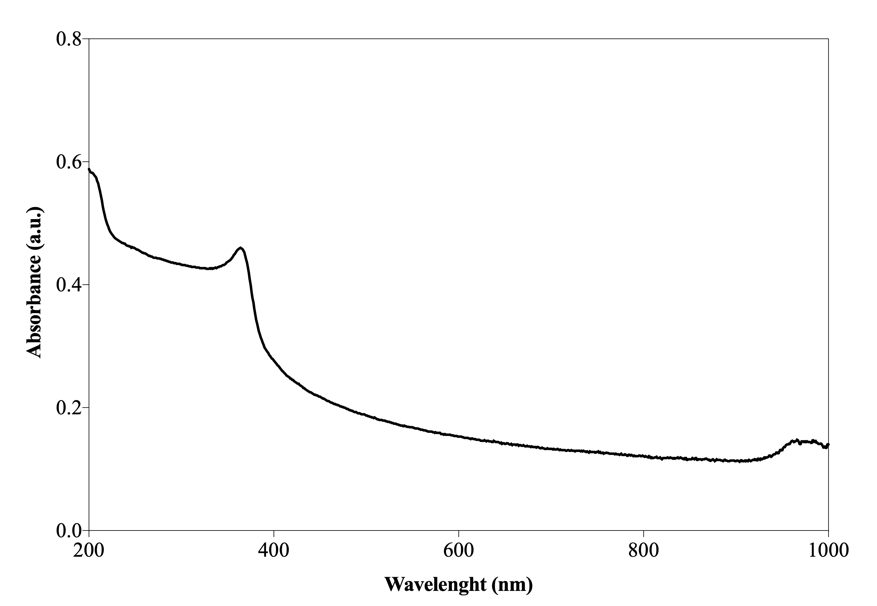


**Figure S-3.** The UV-vis absorbance spectrum of zinc oxide nanoparticles from 200 nm to 1000 nm

Pressure inside 24-well during US irradiation


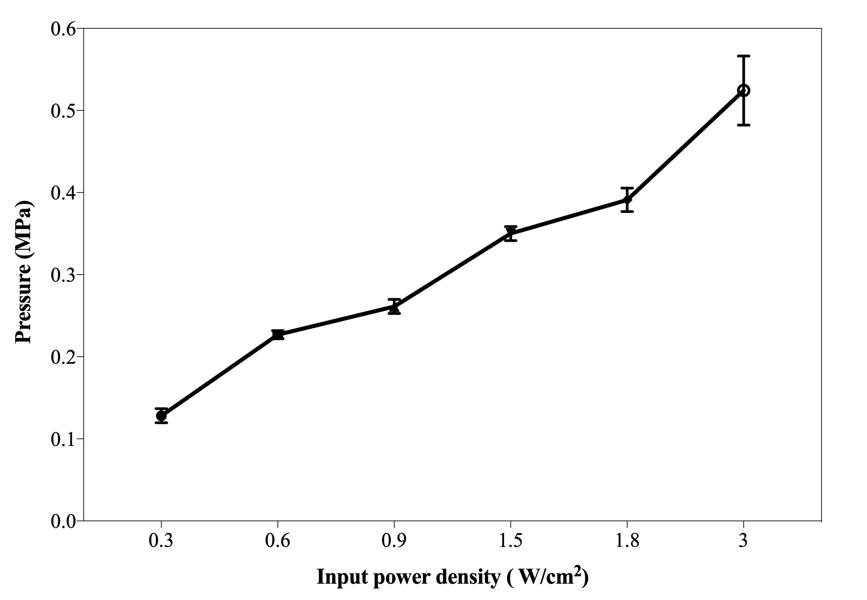


**Figure S-4.** Pressure inside the 24-well at different input power conditions, measured with Ago Hydrophone SN2195 under the ultrasound stimulation by Lipo0 G39. Test were performed in triplicate, at 1 MHz and 100%DC.

Temperature increase during US irradiation inside the 24-well


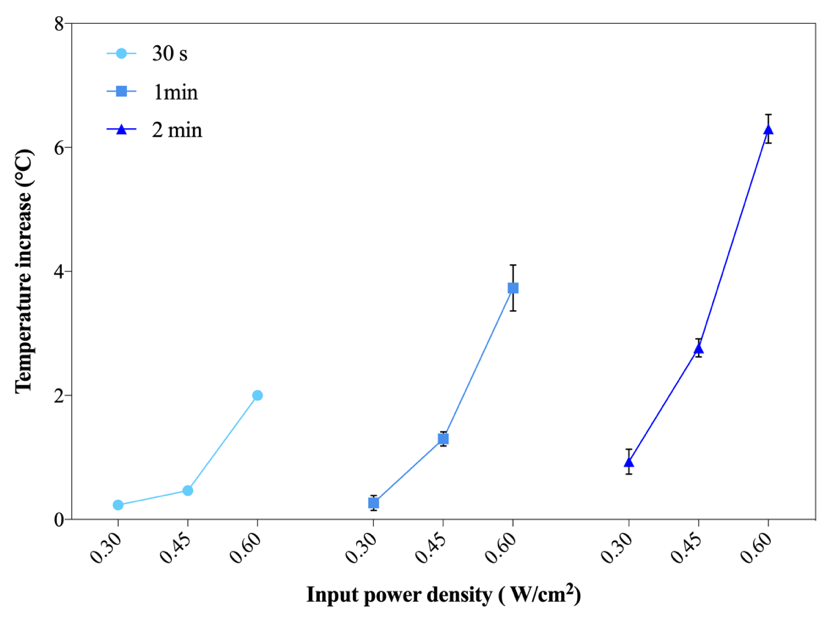


**Figure S-5.** Temperature increase (ΔT) in the 24well during US insonation at different input conditions and exposure times, measured with thermocouple. Test were performed in triplicate, at 1 MHz and 100%DC.

Statistical analyses


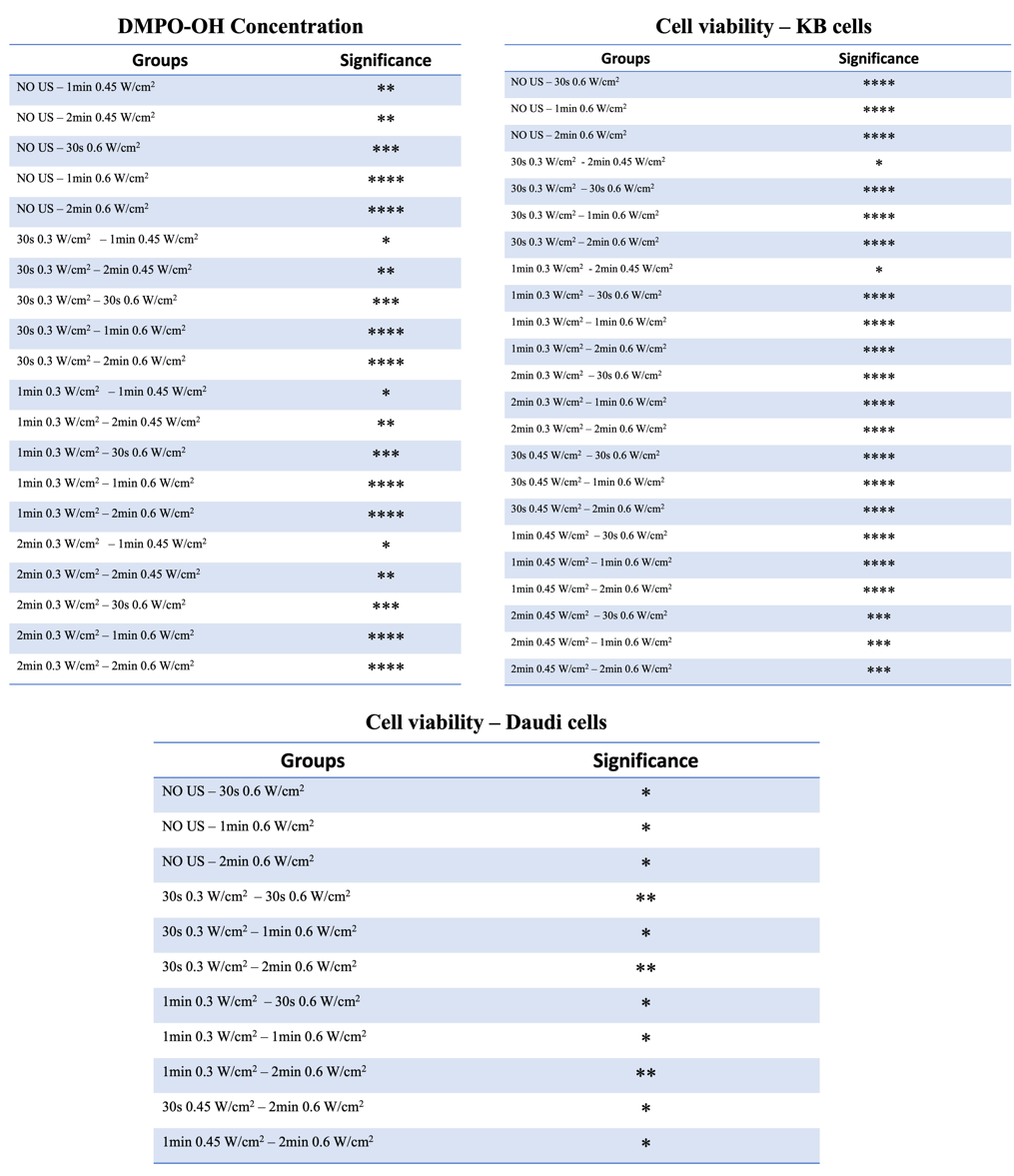


**Figure S-6.** Statistical analys of Figure 4. One-way anova was performed on data sets. . * p<0.0332, ** p<0.0021, *** p<0.0002, **** p< 0.0001


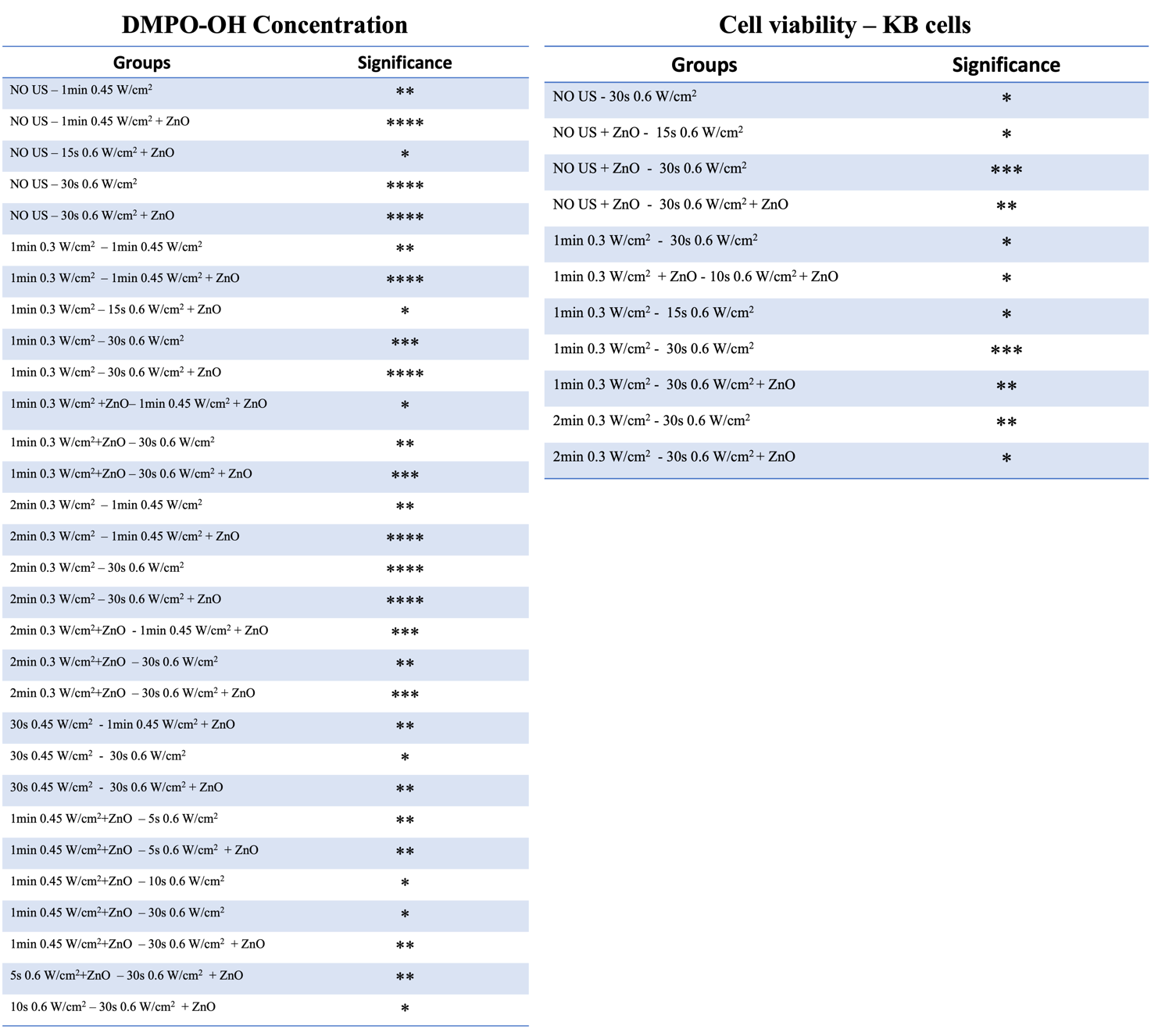


**Figure S-7.** Statistical analys of Figure 5. One-way anova was performed on data sets. . * p<0.0332, ** p<0.0021, *** p<0.0002, **** p< 0.0001
